# Emerging Biological Importance of Central Nervous System Lanthionines

**DOI:** 10.3390/molecules15085581

**Published:** 2010-08-13

**Authors:** Kenneth Hensley, Kalina Venkova, Alexandar Christov

**Affiliations:** 1 Department of Pathology, University of Toledo Medical Center, 3000 Arlington Ave, 43614 Toledo, OH, USA; 2 Department of Neurosciences, University of Toledo Medical Center, 3000 Arlington Ave, 43614 Toledo, OH, USA

**Keywords:** lanthionine, glutathione, transsulfuration, kynurenine, LanCL, CRMP2

## Abstract

Lanthionine (Lan), the thioether analog of cystine, is a natural but nonproteogenic amino acid thought to form naturally in mammals through promiscuous reactivity of the transsulfuration enzyme cystathionine-β-synthase (CβS). Lanthionine exists at appreciable concentrations in mammalian brain, where it undergoes aminotransferase conversion to yield an unusual cyclic thioether, lanthionine ketimine (LK; 2*H*-1,4-thiazine-5,6-dihydro-3,5-dicarboxylic acid). Recently, LK was discovered to possess neuroprotective, neuritigenic and anti-inflammatory activities. Moreover, both LK and the ubiquitous redox regulator glutathione (γ-glutamyl-cysteine-glycine) bind to mammalian lanthionine synthetase-like protein-1 (LanCL1) protein which, along with its homolog LanCL2, has been associated with important physiological processes including signal transduction and insulin sensitization. These findings begin to suggest that Lan and its downstream metabolites may be physiologically important substances rather than mere metabolic waste. This review summarizes the current state of knowledge about lanthionyl metabolites with emphasis on their possible relationships to LanCL1/2 proteins and glutathione. The potential significance of lanthionines in paracrine signaling is discussed with reference to opportunities for utilizing bioavailable pro-drug derivatives of these compounds as novel pharmacophores.

## 1. Introduction

The electronic structure of sulfur imbues sulfurous organic compounds, including sulfurous amino acids, with chemical reactivities beyond those of oxygen-containing analogs. Biosulfur compounds are much more nucleophilic and acidic than oxygen analogs, allowing thiols (RSH) and thioethers (R-S-R') to participate in a far greater range of electrophilic substitutions than the corresponding alcohols (ROH) or ethers (ROR') [1,2]. Also, because sulfur is an element of the third row of the periodic table, it has access to five empty 3d orbitals which can participate in bonding, thus allowing access to valence states beyond those possible for oxygen [1,2]. Bond strength differences between biological sulfur and oxygen containing compounds have another profound effect on the redox biology of sulfurous species: Because S-S and S-O bonds are much stronger than O-O bonds, disulfides and sulfenic acids (RSOH) are more stable than peroxides (ROOR') [1,2]. All these considerations begin to explain why biological systems have evolved to utilize cysteine (Cys) and methionine (Met) for crucial structural, catalytic, and metabolic functions. Free sulfurous amino acids or small peptides are equally crucial to support homeostasis and signal transduction within organisms that live in an oxidizing atmosphere. For example, glutathione (γ-glutamyl-cysteine-glycine, GSH) maintains intracellular compartments in a chemically reduced state [3]; assists to transport amino acids into and xenobiotics out of, tissue parenchyma [3,4]; and helps transduce signals from cell surface receptors to intracellular protein targets via the redox process of reversible glutathionylation [5,6]. Evolution has further exploited sulfur reactivity to permit anabolic pathways of methyl transfer through the high energy intermediate S-adenosyl methionine (SAM) [7]; and to create specific diffusible paracrine substances important to inflammation, as in the case of cysteinyl leukotrienes (LTC_4_, LTD_4_, LTE_4_) [8]. 

The biochemical versatility of organosulfur would suggest that other functions of sulfurous amino acids might exist, with ramifications to health and pathology that remain incompletely appreciated. The purpose of this review is to discuss the amino acid lanthionine (Lan, or alanine-S-alanine) along with some of its derived metabolites, particularly the cyclic thioether lanthionine ketimine (LK; 2*H*-1,4-thiazine-5,6-dihydro-3,5-dicarboxylic acid), which are present in mammalian central nervous system (CNS). Traditional thinking about the origin of Lan as an accident of promiscuous sulfur metabolism is discussed and challenged in light of emerging evidence that LK possesses distinctive biological activities suggestive of a paracrine signaling molecule. This review of lanthionine biochemistry will lead to an introduction of recently discovered lanthionine cyclase-like (LanCL) proteins, with speculations regarding the possible relationship between mammalian LanCLs and lanthionines.

## 2. Lanthionine is an Alternative Product of the Transsulfuration Enzyme CβS, and a Substrate for Subsequent Transamination Reactions

**Scheme 1:**
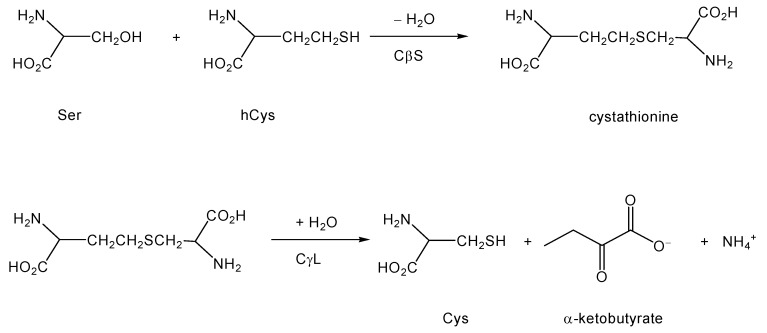
The classic transsulfuration pathway.

During the normal functioning of the folate cycle, methionine is activated by adenosylation to form the high energy intermediate S-adenosyl-methionine (SAM). SAM donates its activated methyl group to appropriate substrates in the course of biosynthetic events. The byproduct of these methyl transfer reactions is S-adenosyl-homocysteine, which hydrolyzes to form homocysteine (hCys; Scheme 1). Homocysteine is not proteogenic and cannot be used directly to make useful substances like GSH, hence must be scavenged, or else its sulfur content would be lost. Additionally, hCys may be directly neurotoxic [9]. Thus, the transsulfuration pathway has evolved to recover useful cysteine from potentially harmful hCys waste. In this classic pathway [7], hCys is first condensed with serine (Ser) via the pyridoxal phosphate (PLP or vitamin B6)-dependent enzyme cystathionine β-synthase (CβS). The product, cystathionine, is cleaved by a second PLP enzyme, cystathioninase (cystathionine γ-lyase, CγL) to yield homoserine (and subsequently, α-ketobutyrate), NH_4_^+^, and Cys (Scheme 1).

**Scheme 2:**
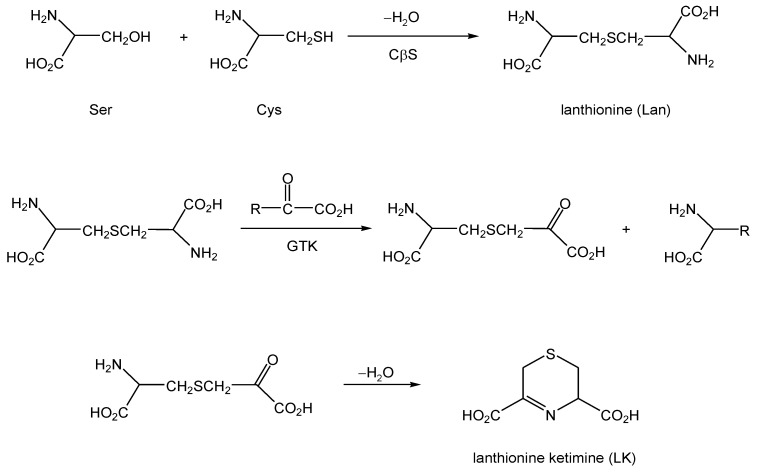
Formation of lanthionine (Lan) through alternative reactions of the transsulfuration pathway, and subsequent conversion to lanthionine ketimine by action of GTK (KAT1). Lan may also form by the action of CβS upon two molecules of Cys, in which case H_2_S is liberated [11].

A less well-appreciated conversion also can be catalyzed by CβS. In broken cell and tissue preparations at least, CβS can catalyze the condensation of Cys (rather than hCys) with Ser or another Cys [10,11]. The product of such reactions is lanthionine (Lan; Scheme 2) rather than cystathionine, as would be the case with the traditional first step of transsulfuration. 

**Figure 1:**
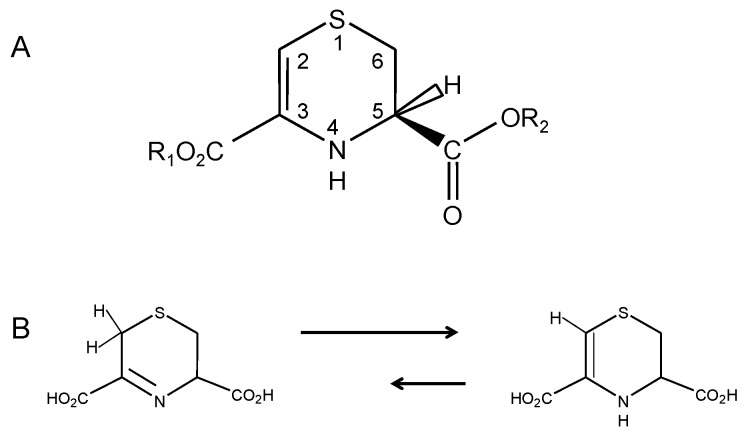
A: Structure and numbering convention for lanthionine ketimine (LK) tautomers and synthetic derivatives. For natural lanthionine ketimine, R_1 _= R_2 _= H. B: Tautomerism between the imine and ene-amine forms of LK.

For years, the existence of alternative reaction pathways accessible to CβS has been largely neglected under the implicit assumption that these pathways represent unproductive avenues or waste products of a promiscuous enzyme. This thinking has been questioned by prescient work from the laboratory of Professor Cavallini in the late 1980s-1990s, and his contemporaries, who noted that Lan could act as a substrate for transaminases, particularly the PLP-dependent enzyme glutamine transaminase K (GTK) also known as kynurenine aminotransferase-1 (KAT1) [12,13,14,15]. GTK catalyzes the transamination of numerous amino acid substrates, including Lan, with α-keto acids (Scheme 2). The products of this chemistry include an intermediate that rapidly cyclizes to yield a thioether ketimine (Scheme 2). When Lan is the aminoacyl substrate for the transamination, the product is specifically lanthionine ketimine (LK) (Scheme 2, Figure 1). The substitution of substrates besides Lan (e.g. cystathionine) gives rise to a family of unusual cyclic thioethers that are demonstrably present in mammalian brain [13]. The same products can be formed through a mechanism catalyzed by amino acid oxidase (AAO), however the transaminase activity is probably a more significant pathway in the mammalian brain owing to the low amount of AAO in brain and its high pH optimum [13,14,15].

There is reason to inquire whether lanthionine conversion to LK is a functional metabolic transformation. The tryptophan oxidation product kynurenine undergoes transamination to yield the cyclic product kynurenic acid (KYNA), which somewhat parallels lanthionine conversion to LK [16]. 

Despite GTK being synonymous with KAT1, kynurenine → KYNA transformation probably does not appreciably proceed through GTK because the K_m_ of GTK for kynurenine is high (4 mM) whereas ambient brain kynurenine levels are very low and glutamine levels are much higher (mM levels) [17]. Nonetheless, KYNA is present in brain where it is now established to be an endogenous anti-excitotoxin that binds to an allosteric site on NMDA receptors to antagonize excessive glutamate neurotransmission [18]. Endogenous KYNA regulates sensitivity of striatal neurons to quinolate, which has implications to the etiology of Huntington’s disease (HD), a condition in which these neurons are particularly vulnerable [19,20]. Thus, KYNA is indicated as an endogenous paracrine substance with important physiological functions still being elucidated. It is therefore conceivable that LK and its molecular family members, formed from similar transamination, might serve paracrine functions. Such hypothesis is aesthetically appealing from the standpoint of metabolic economy, because it replaces the presumptively wasteful promiscuity of both CβS and GTK with functional activities, while simultaneously making use of what might otherwise become a wasteful sulfur leak. In order to become credible, such a hypothesis would require experimental validation and corroboration including the identification of specific, potent, and healthful biological activities inherent to LK or other members of its molecular family of cyclic thioether ketimines. Recently, scientific investigations have begun uncovering just such activities.

## 3. Lanthionine Metabolites and Their Synthetic Derivatives Display Antioxidant, Anti-Inflammatory, Neuroprotective and Neurotrophic Activities

Very little research lately has focused on physiological roles for Lan and LK, though such roles have been long hypothesized by some biochemists. Using HPLC and gas-liquid chromatography, Cavallini and colleagues performed seminal work in this area by measuring LK in mammalian brain at concentrations near 1 nmole/g tissue [13,21,22]. These investigators reported that [^35^S]LK bound synaptosomal membranes tightly (K_d_ = 58 nM, in the range of typical neurotransmitter affinities) [23]. [^35^S]LK binding was saturable and reversible with unlabeled LK, suggesting a receptor: ligand interaction [13,23]. The binding was specific because chemically reduced LK (thiomorpholine dicarboxylic acid) could not displace [^35^S]LK [13,23]. Such binding would seem to suggest the existence of specific LK binding partners and hence, possible inherent LK activities.

Recent studies have demonstrated unexpected bioactivities of LK that are observable at low micromolar concentrations in cell culture systems [24] or *in vivo*. For example, we synthesized cell permeable LK derivatives including hydrolysable LK-5-ethyl ester (LKE) from reaction of 3-bromopyruvate with L-cysteine ethyl ester [24,25]. Both LK and LKE suppressed nitric oxide (•NO) synthesis in tumor necrosis factor-α (TNFα) or lipopolysaccharide (LPS)-challenged microglia and macrophages, with the ester being more potent [24,25]. Furthermore, LKE protected NSC-34 motor neuron-like cells from toxicity of hydrogen peroxide (H_2_O_2_) [24,25] and from toxicity of microglia-conditioned medium [25]. Most notably, LKE promoted the growth factor-stimulated outgrowth of neurites in NSC-34 cells [24,25] which could have broad implications to the use of synthetic LK derivatives or tissue-penetrating pro-drugs for the treatment of neurodegenerative conditions [24,25].

**Figure 2:**
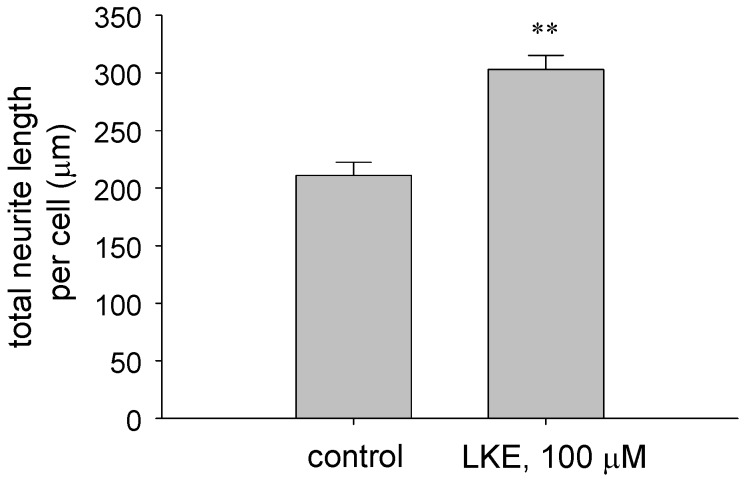
LK-5-ethyl ester (LKE) promotes neurite extension in primary, dissociated chick dorsal root ganglia (DRG) cultures. Neurons were treated with LKE or saline vehicle for 24h and quantitatively assessed for neurite morphometry as described in the text.

We have expanded on these findings through further cell culture investigations and in the study of a murine model for the motor neuron disease amyotrophic lateral sclerosis (ALS). Whereas NSC-34 cells are immortalized and may not accurately depict the cell physiology of a primary neuron, studies were performed to assess LKE effects on neuritigenesis in primary dissociated chick dorsal root ganglia (DRG) cultures prepared essentially as described by Margiotta and Howard [26]. Cultures were treated with LKE at 2-3 days *in vitro* (DIV), and neuron morphometry was quantified from bright field micrographs by a blinded observer using Metamorph® software (Molecular Devices, Sunnyvale CA, USA). At 100 μM, LKE significantly increased the mean neurite length of dissociated DRG neurons (Figure 2), similar to effects observed previously in NSC-34 cultures [24].

**Figure 3:**
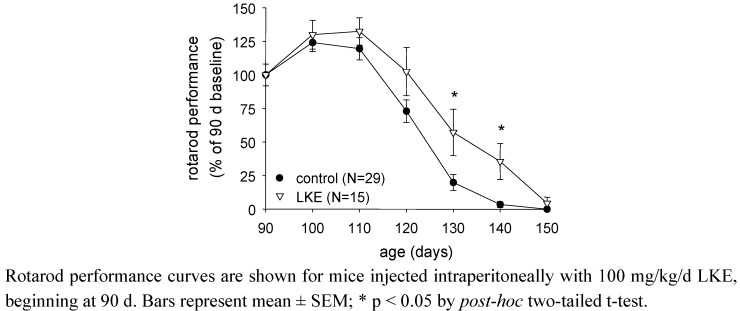
LKE slows progression of paralytic disease in the SOD1^G93A^ mouse model of familial amyotrophic lateral sclerosis (ALS).

Subsequent studies have begun to explore effects of LKE *in vivo*, as a pharmacological candidate. In a first effort to assess LKE effects against a model of spontaneous neurodegeneration, SOD1^G93A^ mutant mice modeling familial ALS [27,28] were treated with intraperitoneal (i.p.) injections of 100 mg/kg/d LKE in saline vehicle, from 90 d throughout the remaining mouse lifespan. LKE is relevant to test in this mouse because the SOD1^G93A^ mouse experiences robust neuroinflammation concomitant with TNFα-driven microglial activation, oxidative stress, and significant progressive distal axonopathy [27], all of which might be expected to be mitigated by LKE based on the ester’s observed effects in cell culture. Mouse motor function was assessed as previously described using a standard rotarod test [27,28], at sequential 10 d intervals (Figure 3). Specific groups of mice were treated with LKE in saline or saline vehicle only. As illustrated in Figure 3 and Table 1, LKE significantly slowed progression of clinical motor neuron disease [25], mostly through delaying onset of clinical paralysis as defined by a clinical leg-splay test [27,28]. Overall survival was increased by LKE based on logrank analysis (Table 1). These findings suggest that further research is justified to determine the potential of bioavailable LK derivatives or pro-drugs for the treatment of neurodegenerative pathologies.

**Table 2:** Effects of LKE on clinical parameters in SOD1^G93A^ mice.

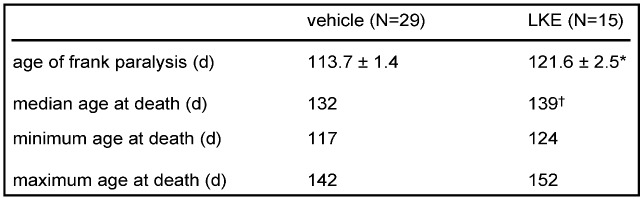

Drug was administered intraperitoneally, in saline, at 100 mg/kg/d beginning at 90 d. Data are mean ± SEM. * p < 0.05 by t-test; ^† ^p < 0.05 by log-rank test.

## 4. Affinity Proteomics Investigations Reveal Lanthionine Ketimine Interaction with Select Subsets of Mammalian Brain Proteins: Implications for Relevance to Neurological Disease

Work by Fontana, Cavallini and colleagues [23] suggested that LK might bind selectively to brain proteins, but candidate proteins were not identified in this early pioneering research. Recently we took advantage of modern mass spectrometry-assisted protein microsequencing techniques to screen a mammalian brain proteome for LK binding partners [24]. LK was coupled to a solid phase support and exposed to bovine brain protein extract. Free LK was used to selectively elute LK-bound species. Three proteins emerged that appeared to bind LK selectively but were not likely to bind nonselectively to uncoupled solid phase supports. These were collapsin response mediator protein-2 (CRMP2; also known as dihydropyrimidinase-like protein-2 (DRP2) or DPYSL2); syntaxin-binding protein-1 (STXBP1, also known as Munc18 or nSec1); and lanthionine synthetase-like protein-1 (LanCL1) [24]. Other proteins including myelin basic protein and proteolipin, which are important components of oligodendrocytes and myelin sheaths, also displayed evidence for LK interaction, though some nonspecific binding of these basic proteins to the affinity matrix could not be excluded [24]. The fact that LK selectively bound LanCL1 in an unbiased, brain proteome-wide affinity screen strongly suggests a functional relationship of the small molecule to the protein, and argues against the two entities being related merely by an accident of nomenclature.

CRMP2, STXBP1, and LanCL1 proteins are becoming recognized for their roles in neurite outgrowth, synaptogenesis, and neurotransmission. CRMP2/DRP2/DPYSL2 is crucial to mediate growth factor-dependent axon and dendrite growth and regulates neuron polarization (the process by which one neurite becomes an axon) during embryonic neurogenesis [29]. STXBP1/Munc-18/nSec1 is part of the presynaptic protein machinery that regulates fusion of neurotransmitter vesicles with the plasma membrane during neurotransmitter docking and exocytosis [30]. LanCL1 is a particularly intriguing protein that also binds GSH [31,32]. Cellular functions of LanCL1 and its homolog LanCL2 are still being elucidated (discussed further, below) but mutants of LanCL1 appear to act in a dominant negative fashion to interfere with growth factor-dependent neurite outgrowth in neuroculture [32]. The documented relevance of both CRMP2 and LanCL1 to neuritigenesis therefore may begin to suggest a basis for the observed neurotrophic or neuritigenic effects of LK (described above). Other brain protein binding partners could possibly exist that were not effectively canvassed by the proteomics techniques thus far applied to their discovery. Cell surface membrane receptors and strongly membrane associated proteins, in particular, likely would not have been identified in our previous investigations.

Emerging proteomics studies are suggesting an association of LK-interacting proteins with certain neurological conditions. For instance, MBP and CRMP2/DPYSL2 were previously found differentially expressed in human schizophrenia brain tissue [33], as well as lanthionine synthetase-like protein [34]. It remains to be determined whether such associations of LK-binding proteins are pathogenically relevant or only epiphenomena. Since neuroinflammation has been implicated as a contributing factor to schizophrenia [35], and LK has demonstrable anti-neuroinflammatory actions (discussed above), future studies might be justified to explore lanthionine levels in schizophrenia, or perhaps even use in disease palliation. 

## 5. Eukaryotic Lanthionine Cyclase-Like Proteins (LanCL1 and LanCL2): Functionally Connected to Brain Lanthionines, or Merely Related through an Accident of Nomenclature?

In 2007, our lab identified lanthionine synthetase-like protein-1 (LanCL1) as a prominently expressed brain protein that strongly bound both GSH and GSSG (but not other sulfurous amino acids such as cysteine) and which was over-expressed in spinal cords of SOD1^G93A^ mice [31]. LanCL1 subsequently was crystallized with bound GSH and the X-ray structure published [32]. The existence of LanCL1 (and its N-terminally myristoylated homolog LanCL2) begs the question of whether LanCL1/2 is functionally related to either Lan or LK, or whether the relationship is merely one of accidental nomenclature. Some elaboration on this issue is justified.

As yet, LanCL1/2 have no reported enzymatic functions, but are sequence homologs of bacterial lanthionine cyclase (LanC) enzymes which catalyze regioselective, intramolecular conjugation of protein Cys residues to nearby, dehydrated Thr or Ser residues within specific precursor polypeptides [36,37]. In most cases the relevant Ser or Thr is dehydrated to dehydroalanine (Dha) or dehydro-butyrine, respectively, by a separate dehydratase which does not have a mammalian homolog [36]. The resulting product of multiple LanC catalyses is a polymacrocyclic polypeptide termed a lantibiotic [36,37]. Lantibiotics are amongst the most potent antibiotic substances yet discovered, and as such, have generated tremendous research interest amongst microbiologist and medicinal chemists [36,37]. Both LanC and LanCL1 have a structurally similar Zn^2+^ binding domain, which in LanC is the catalytic pocket for the enzyme. Glutathione binds in the LanCL1 version of this pocket, tightly associating with specific amino acid residues to constrain GSH orientation while allowing the central thiol to ligate the Zn^2+^ metallocenter [32]. Ample space exists within the GSH binding pocket of LanCL1 to permit co-occupancy of another molecule [32], but as of yet no co-substrate has been identified for GSH conjugation by LanCL1. Given the homology between bacterial LanCs and mammalian LanCL1, and the fact that both enzymes dock with cysteinyl peptides in a similar configuration, the eukaryotic protein may have inherited a similar sulfur catalytic chemistry to the prokaryotic enzyme.

These coincidences alone do not prove or even strongly suggest that CNS lanthionines are functionally related to LanCL1/2. On the other hand, the empirical fact that immobilized LK selectively binds LanCL1 within the milieu of a whole mammalian brain proteome extract [24] strongly suggests a meaningful binding relationship, and therefore a likely functional relationship, between LK and LanCL1. 

If lanthionyl compounds do prove functionally related to LanCL1/2, the relationship could be that of allosteric mediation or that of an enzyme and its catalytic product. Bassaganya-Riera and colleagues recently described a molecular modeling study in which both the natural metabolite abscissic acid (ABA) and synthetic thiazolidinediones (TZDs, a class of insulin sensitizing medicaments approved for type 2 diabetes) appeared to bind to LanCL2, at a site distinct from the metallocenter [38]. Following reports by Sturla *et al*. [39] that ABA activates TZD-sensitive peroxisome proliferation activated receptor-gamma (PPARγ) indirectly through LanCL2, Bassaganya-Riera *et al*. speculate that the ABA interaction with LanCL2 represents an allosteric-type interaction upstream from PPARγ [38]. Based on this precedent, an allosteric binding of LK to LanCL1/2 cannot be excluded.

**Scheme 3:**
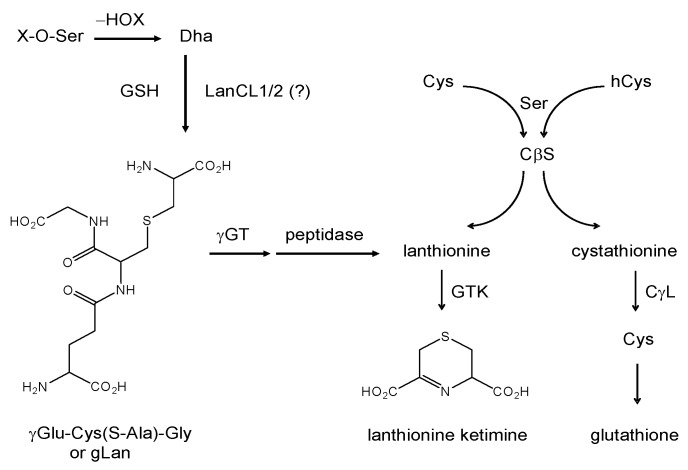
Summary of known and hypothesized routes for enzymatic formation of lanthionine ketimine, via either the transsulfuration and aminotransferase pathways, or through a hypothetical glutathione-lanthionine (gLan) intermediate. As of the publication of this review, gLan has not been found naturally in biological tissues. The involvement of LanCL proteins in gLan synthesis is theoretically possible. If gLan is formed *in vivo*, Lan would be expected to form upon exposure to ambient γ-glutamyl transpeptidase (γGT) and carboxypeptidase. Lanthionine conversion to LK then could occur through GTK. Dha = dehydroalanine.

If the relationship between LanCL1/2 and lanthionines proves catalytic (with Lan, LK, or related compound as either substrate or product) then implications could exist for lanthionyl derivative use as a type of metabolic replacement therapy for conditions wherein LanCL1/2 function is insufficient. Since mammals do not possess a Ser/Thr dehydratase, it is unlikely that GSH would frequently react through Michael addition to an enzymatically-generated peptidic alkene. On the other hand, protein-bound dehydroalanine (aminoacrylate) has been reported in long-lived mammalian proteins where it is thought to form through base catalyzed dehydration of phosphoserine residues [40] but can also form through *S*-nitrosocysteine decomposition under certain circumstances [41]. LanCL1/2 thus might conjugate GSH to specific post-translationally modified peptides (yet to be discovered). A LanCL1/2 catalyzed β-elimination reaction between GSH and phosphoserine or even phosphatidylserine (PtdSer) might be considered as a thermodynamically plausible route to formation of lanthionine within the context of a glutathione backbone (Scheme 3)**. ** The hypothetical glutathione-lanthionine chimera (*i.e*., glutathione-*S*-(β-alanine) or “gLan”, Scheme 3) could be metabolized to free Lan and hence to LK through known pathways alluded to in the preceding discussions (see Scheme 3).

**Figure 4:**
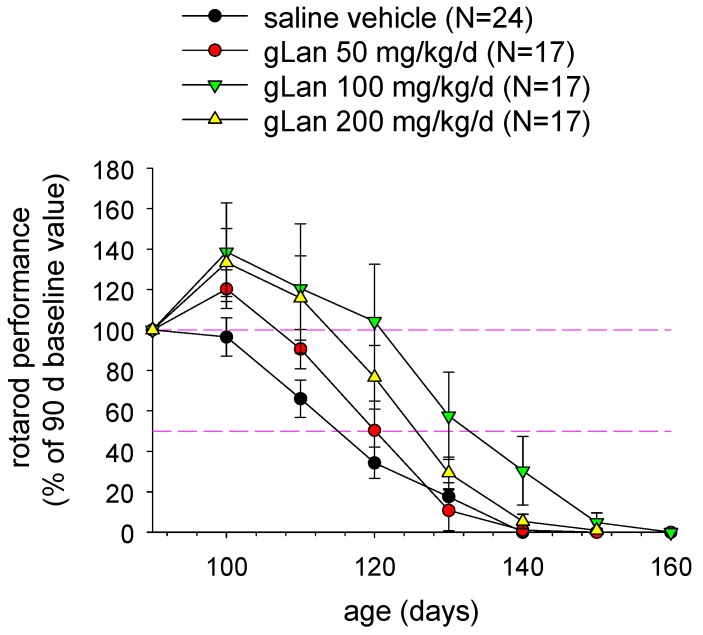
Treatment with synthetic gLan improves motor function in SOD1^G93A^ mice. Mice were treated with daily intraperitoneal injections of gLan, at the indicated dose, 5 days/ week beginning at 90 d, and motor performance was evaluated by rotarod task as described in the text. Data points indicate mean ± SEM.

We have synthesized gLan through the reaction of GSH with β-chloroalanine and we are actively investigating its chemical and biological properties and potential as a novel medicament. If gLan is a metabolic precursor for LK then gLan would be anticipated to display bioactivities similar to those of LK. Accordingly, an experiment was conducted to treat SOD1^G93A^ mice with intraperitoneal gLan similar to the study of LK described above. Mice were injected with synthetic gLan in saline at 50-200 mg/kg/d, five days per week (Monday-Friday) beginning at 90 d of age. Mice were assessed at baseline and every 10 d for motor functional ability using the rotarod task as described previously [27,28]. As illustrated in Figure 4 and Table 2, gLan dose-dependently improved motor function in SOD1^G93A^ mice up to 110 d or 120 d with 100 mg/kg being optimum (p < 0.01 for overall gLan effect based on repeated measures ANOVA). Interestingly, average rotarod performance at 100 d and 110 d in mice treated with 100-200 mg/kg/d gLan, actually improved above the 90 d baseline value (Figure 4). Transgenic animals treated with these doses of gLan displayed, on average, twice the motor function as saline treated control animals at ages <120 d (Figure 4). Motor function dropped off rapidly amongst all groups at ages > 110 d (Figure 4). As in the case of LKE treatment, chronic intraperitoneal gLan at 100-200 mg/kg/dose tended to increase the age at which onset-of-paralysis was observed (based on a leg-clinch test [28]) and the mean age of death of the SOD1^G93A^ mice (Table 2). When motor performance curves were interpolated to estimate the age at which mice lost an average 50% of baseline motor function, the 100 mg/kg/dose gLan treatment group reached this clinical endpoint approximately 15 d later than the vehicle-treated group (Figure 4). To our knowledge this is the first data indicating possible beneficial bioactivities inherent to the gLan molecule.

**Table 2:** Effects of gLan upon onset-of-paralysis and age-at-death parameters in SOD1^G93A ^mice treated with the indicated daily dose of gLan beginning at 90 d as indicated in text and Figure 4. * p < 0.02 by two-tailed t-test.

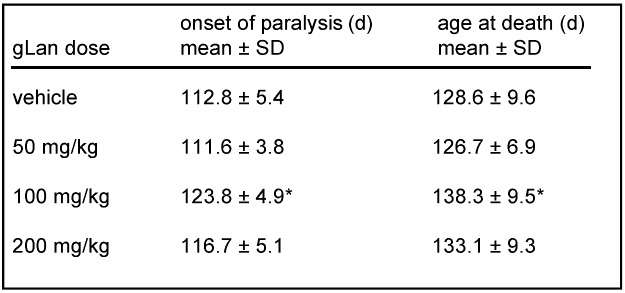

## 6. Summary

Lanthionine (Lan) exists in mammals as the free amino acid but can also exist as a crosslinking amino acid within polypeptides or in the context of glutathione-S-alanine (gLan). The existence of lanthionine in mammalian brain has been known for years but is gaining more interest due to recent discoveries of neuroprotective and anti-inflammatory properties inherent to its metabolite lanthionine ketimine; and due to recent research interest in mammalian LanC-like proteins, that are homologous to bacterial enzymes responsible for prokaryotic lantibiotic syntheses. Taken together these recent findings begin to suggest that central nervous system lanthionyl compounds may be physiologically functional molecules rather than mere metabolic waste. Research into the origin, function, and pharmacological potential for both natural and synthetic lanthionines is still in its early stages. Further work in this area might clarify an underappreciated metabolic junction between transsulfuration and transamination pathways, and might yield new concepts and small molecules for treating both metabolic dysfunctions and neurodegenerative diseases.
